# An Integrative Study of Mycobiome in Different Habitats from a High Arctic Region: Diversity, Distribution, and Functional Role

**DOI:** 10.3390/jof9040437

**Published:** 2023-04-03

**Authors:** Xiufei Chen, Dong Yan, Liyan Yu, Tao Zhang

**Affiliations:** 1 China Pharmaceutical Culture Collection, Institute of Medicinal Biotechnology, Chinese Academy of Medical Sciences & Peking Union Medical College, Beijing 100050, China; 2 Xinxiang Key Laboratory of Pathogenic Biology, Department of Pathogenic Biology, School of Basic Medical Sciences, Xinxiang Medical University, Xinxiang 453003, China

**Keywords:** fungal diversity, high arctic, habitat specificity, functional role

## Abstract

In the Arctic ecosystems, fungi are crucial for interactions between soil and plants, the cycling of nutrients, and the transport of carbon. To date, no studies have been conducted to thoroughly examine the mycobiome and its functional role in various habitats of the High Arctic region. The aim was to unravel the mycobiome in the nine habitats (i.e., soil, lichen, vascular plant, moss, freshwater, seawater, marine sediment, dung, and marine alga) in the Ny-Ålesund Region (Svalbard, High Arctic) using a high-throughput sequencing approach. A total of 10,419 ASVs were detected. Among them, 7535 ASVs were assigned to unidentified phyla, while the remaining 2884 ASVs were assigned to 11 phyla, 33 classes, 81 orders, 151 families, 278 genera, and 261 species that were known. The distribution of the mycobiome was driven by habitat specificity, indicating that habitat filtering is a crucial factor influencing the fungal assemblages at a local scale in this High Arctic region. Six growth forms and 19 fungal guilds were found. The ecological guild (e.g., lichenized, ectomycorrhizal) and growth form (e.g., yeast, thallus photosynthetic) varied significantly among various habitats. In addition, the occurrence of 31 fungal species that are considered to be potential pathogens was determined. These results will increase our understanding of fungal diversity and its functional significance in this distinctive High Arctic area and thereby establish the groundwork for prediction about how the mycobiome will alter in various environments as a result of anticipated climate change.

## 1. Introduction

Global climate change is causing the Arctic to warm at unprecedented rates, which has a significant impact on the Arctic ecosystems by altering weather patterns, lowering sea ice cover, melting massive areas of permafrost soil, and changes in Arctic vegetation [1,2,3]. The Arctic tundra, which makes up around 5% of the planet’s land area, is one of the harshest habitats on earth due to its low temperatures, repeated freeze-thaw cycles, wet-dry cycles, and lack of organic matter. Up to now, understanding the effects of warming on the Arctic ecosystem has been a difficult task. The Arctic has a variety of habitats that are home to numerous potentially viable, mainly uncharacterized microorganisms. Warming has been demonstrated to change the composition of the microbial communities in Arctic soil, including bacteria [4] and fungi [4,5,6]. Therefore, to predict how Arctic ecosystems will respond to rising temperatures, it is crucial to have a thorough understanding of the region’s microbiome [7].

The majority of earlier studies used conventional isolation techniques to investigate the diversity of cultivated fungi in Arctic habitats (e.g., soils [8,9], lichens [10], freshwater [11], and cryoconite holes [12]). Due to their selectivity, the cultivation-based approaches, however, probably do not accurately reveal the diversity of the mycobiome. Numerous studies have recently used targeted amplicon sequencing (i.e., Roche 454 pyrosequencing, Illumina sequencing) to characterize the diversity and composition of the mycobiome in the Arctic, including those found in the soils [13,14,15,16], marine sediments [17], lichens [18], mosses [16,19], vascular plants [16,20,21,22], seawater [23,24], and freshwater [16,25,26]. However, there are no comprehensive studies that unravel and compare the diversity and distribution of the mycobiome in various habitats from the High Arctic region. The fungal assemblages at the local scale of the High Arctic may be impacted by habitat overlap or specialization, but this is still unclear.

In the Arctic, shifts in the composition of fungal functional groups are considered to have an impact on permafrost carbon storage, ecosystem respiration, and nutrient cycling [27]. The ecological role of fungi in the Arctic ecosystem, however, is only partially understood (e.g., soil [13,27,28,29], vegetation [19], and seawater [30]). In addition, some environmental fungi may evolve pathogenic potential as a result of climate warming, and the presence of potential fungal pathogens in Arctic environments (such as snow, subglacial ice, glacial meltwater, and air) has been observed [31]. However, there is still a considerable information gap between the diversity of fungi and their ecological functions in the Arctic ecosystem.

To address this knowledge gap, we used high-throughput sequencing to analyze the mycobiome associated with the nine habitat types (i.e., soil, lichen, vascular plant, moss, freshwater, seawater, sediment, dung, and marine alga) in the Arctic region. The aims of the present study were to (1) comprehensively reveal the diversity and functional role of the mycobiome in various habitats from a High Arctic region; (2) assess how and to what extent the habitat types influence the mycobiome at the local scale. We hypothesized that habitat selection instead of habitat overlap would determine the mycobiome in different habitats of the High Arctic. Knowledge of the diversity and functional role of the mycobiome in the High Arctic will be enhanced by the findings of the present study.

## 2. Materials and Methods

### 2.1. Sampling Area

**Figure 1:**
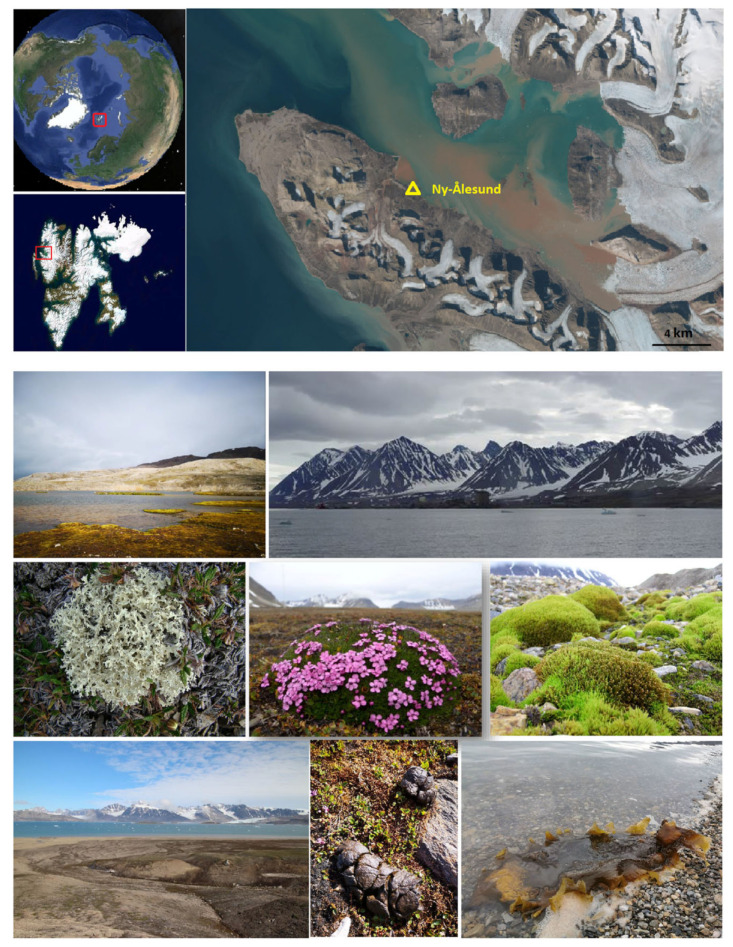
The location of the sampling site and views of various habitats (e.g., soil, lichen, vascular plant, moss, freshwater, seawater, dung, and marine alga) in the Ny-Ålesund Region (Svalbard, High Arctic).

The sampling area was situated in the Ny-Ålesund Region (78°55′ N, 11°56′ E), which lies in the north-western region of Spitsbergen, Svalbard. Svalbard is an Arctic archipelago that lies completely within the High Arctic Zone and is geographically separated from mainland Eurasia. Polar cyclones and the circulation of the North Atlantic Ocean both have an impact on the Svalbard archipelago. According to Maturilli et al. [32], the mean temperatures of the Ny-Ålesund Region range from −17.0 to −3.8 °C in January to 4.6 to 6.9 °C in July. There is no sunset from 18 April to 24 August, and there is no sunrise from 25 October to 17 February (polar night). A total of 114 samples from nine habitats were collected during China’s Arctic expedition in July 2013 (Figure 1, Table S1), comprising 69 samples in the present study (including lichen, marine alga, seawater, marine sediment, dung, and moss) and 45 samples in a previous study (including soil, freshwater, moss, and vascular plant) [16]. The Svalbard Science Forum, the Chinese Arctic and Antarctic Administration (CAA), and the State Oceanic Administration (SOA) of China all provided their permission for the sample collection. Before being flown to our laboratory in China, the samples were frozen at –20 °C in the Yellow River Station (China) for approximately 20 days.

### 2.2. DNA Extraction, PCR Amplification, and Illumina Sequencing

(1) For the samples of marine sediment and dung, a PowerSoil DNA Isolation Kit (MO BIO Laboratories Inc., Carlsbad, CA, USA) was used to extract the DNA from 0.25 g of each sample according to the manufacturer’s instructions. (2) For the samples of freshwater and seawater (1000 mL of each sample), a PowerWater DNA Isolation Kit (MO BIO Laboratories Inc., Carlsbad, CA, USA) was used to extract the DNA from the membrane-bound biomass according to the manufacturer’s instructions. (3) For the samples of lichen, moss, and marine alga, before DNA extraction, the tissues were surface sterilized, as described by Zhang et al. [12]. A PowerSoil DNA Isolation Kit (MO BIO Laboratories Inc., Carlsbad, CA, USA) was then used to extract the DNA from the sterilized tissues, as described by Zhang et al. [18].

The nuclear ribosomal internal transcribed spacer 1 (ITS1) of the fungi was amplified from the acquired DNA using ITS1F (5′-CTTGGTCATTTAGAGGAAGTAA-3′) [33] and ITS2 (5′-GCTGCGTTCTTCATCGATGC-3′) [34] with a 10-nucleotide barcode. PCR amplification was then performed, and the reaction mixture (50 μL) contained the template DNA (about 10 ng), 10× EasyTaq buffer (5 μL), dNTPs (2.5 mM, 2 μL), EasyTaq DNA polymerase (5 units, 1 μL), primers (10 μM, 1 μL), and nuclease-free water. The PCR program began with an initial denaturation step at 95 °C for 2 min, followed by 30 cycles of 95 °C for 30 s, 55 °C for 30 s, and 72 °C for 30 s, and a final extension step at 72 °C for 5 min [18]. Amplicons were extracted, purified, and paired-end sequenced (2 × 300 bp) on an Illumina MiSeq platform by Shanghai Majorbio Bio-pharm Technology Co., Ltd. (Shanghai, China). The negative control samples were subjected to both DNA extraction and PCR. Since no quantifiable DNA was discovered in these negative controls, further sequencing was not performed on them.

### 2.3. Sequencing Data Processing

FLASH software was used to combine the paired-end reads [35]. Subsequently, based on the unique barcodes of each sample, reads were assigned to each one. QIIME 2 version 2022.01 was used to process the unprocessed raw demultiplexed sequences [36]. The DADA2 plugin, a component of QIIME 2, was used to denoise, dereplicate, and filter (for chimeras) the paired-end reads [37]. The bases with quality scores below 35 were removed from the raw reads. The first 26 nucleotides of the 5′ end were removed from both the forward and reverse reads. The forward and reverse sequences both had their 3′ ends cut off at locations 240 and 230, respectively. The read number was set to 100,000 for training the error model. Denovo clustering (100% similarity) was conducted using VSEARCH [38], which is a part of QIIME 2. Amplicon sequence variations (ASVs) were assigned to specific taxa using the classify-sklearn classifier [39] against the UNITE Fungi version 8.3 reference database [40], which has been trained to recognize the fungal ITS1 gene. ASVs with read counts under five were eliminated. The data were then subsampled, with each sample containing 14,866 sequences. Using the FFT-NS-1 method, MAFFT version 7 was used to align with typical ASV sequences, and the Average linkage (UPGMA) method was utilized to build a phylogenetic tree [41].

### 2.4. Statistical Analyses

MicrobiomeAanalyst was used to analyze the data obtained [42]. Non-metric multidimensional scaling (NMDS) ordination showing the mycobiome composition (at the ASV level) in different samples was performed using unweighted UniFrac distance. An analysis of similarity (ANOSIM) was also used to reveal the dissimilarity of the mycobiome among nine habitat types at the ASV level (unweighted UniFrac distances). Dendrogram analysis of the mycobiome in the 114 samples from the nine habitats was performed (average clustering algorithm and unweighted UniFrac distance). The number of fungal ASVs in each of the nine habitats was represented by a Venn diagram. To investigate how different fungal genera interact with one another in different habitats, correlation network analysis was performed using Spearman’s rank correlation test (permutation: 100, *p*-value threshold: 0.01, correlation threshold: 0.70). The functional compositions of the mycobiome were identified using FungalTraits version 1.2 [43]. Using a factorial Kruskal-Wallis test, the distinct taxonomic units (phylum, class, order, family, and genus) and functional roles (guild and growth form) among the nine habitat types were identified using the linear discriminant analysis effect size (LEfSe) analysis. The Atlas of Clinical Fungi was used to determine potential human pathogens [44].

## 3. Results

### 3.1. Sequence Data

From 114 samples, a total of 4,265,338 raw reads were obtained. The raw reads per sample ranged from 19,836 to 105,159 (Table S1). The trimmed reads per sample ranged from 14,896 to 97,477 (Table S2). Following the removal of the uncommon ASVs (<five reads), the total number of reads for 10,453 ASVs in 114 samples was decreased to 3,491,225. After each sequence library was rarefied to 14,866 reads, 1,694,724 reads from 114 samples and 10,419 ASVs were preserved. The ASV number per sample ranged from 2 to 681. Overall, 7535 ASVs were assigned to unidentified phyla, while the remaining 2884 ASVs were assigned to 11 phyla, 33 classes, 81 orders, 151 families, 278 genera, and 261 species that were known (Table S3).

### 3.2. Diversity and Composition of the Mycobiome in the Nine Habitats

**Figure 2:**
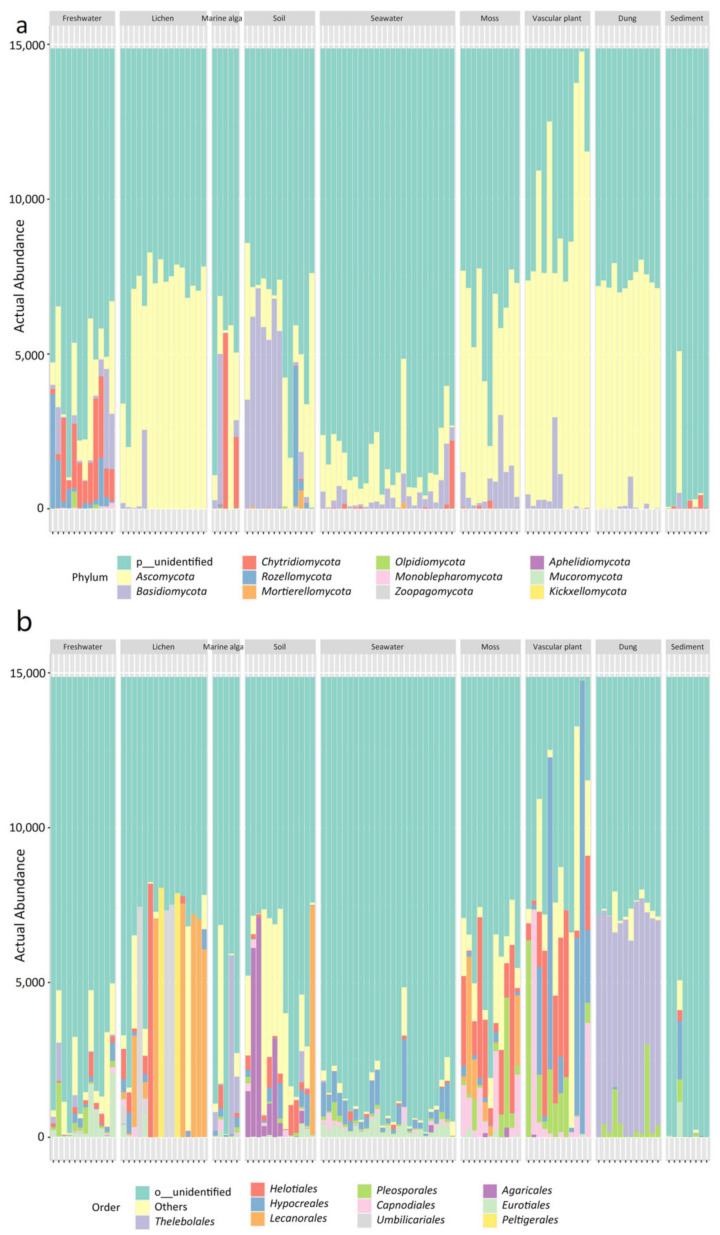
Bar plots showing the abundance of (**a**) fungal phyla and (**b**) fungal orders in the 114 samples collected from nine habitats of the Ny-Ålesund Region (Svalbard, High Arctic).

The nine habitats have different mycobiome compositions, as shown by the stacked bar plots (Figure 2). Reads assigned to unknown phyla predominated in most samples of the nine habitats, especially in seawater and marine sediment. The eleven known phyla included *Ascomycota*, *Basidiomycota*, *Chytridiomycota*, *Rozellomycota*, *Mortierellomycota*, *Olpidiomycota*, *Monoblepharomycota*, *Zoopagomycota*, *Aphelidiomycota*, *Mucoromycota*, and *Kickxellomycota* (Figure 2a). At the order level, *Thelobolales* was more abundant than other orders in the habitat of animal dung, whereas *Hypocreales* was dominant in the seawater habitat. In addition, the order *Agaricales* was abundant in the soil habitat and the order *Helotiales* was abundant in the habitats of moss and vascular plant (Figure 2b).

### 3.3. Dissimilarity of the Mycobiome among the Nine Habitats

**Figure 3:**
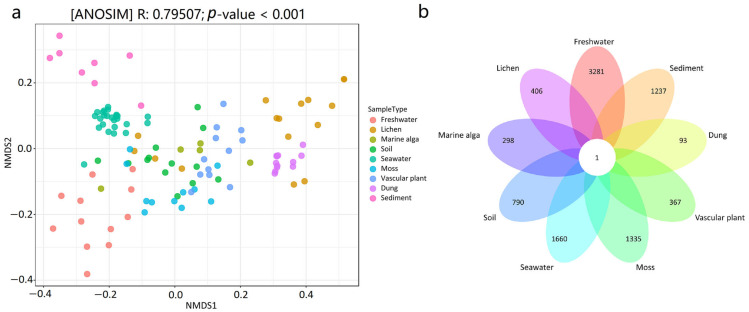
(**a**) NMDS ordination plot showing the spatial pattern of the mycobiome in the 114 samples from the nine habitats, (**b**) Venn diagram showing the number of ASVs in the nine habitats.

The NMDS plot revealed that the mycobiome compositions at the ASV level were different among the habitat types (Figure 3a). The results of the ANOSIM test also revealed that there were significant differences in the mycobiome compositions between the nine distinct habitats (*R* = 0.79507, *p* < 0.001). The results from the pairwise ANOSIM comparisons revealed differences in the mycobiome compositions among various habitats (Table 1). For example, seawater and marine sediment harbored significantly different mycobiome compositions (*R* = 0.90845, *p* < 0.001), and marine sediment and vascular plant harbored significantly different mycobiome compositions (*R* = 1, *p* < 0.001). Only one ASV was shared by all nine habitats, according to the Venn diagram (Figure 3b). The linkages between the mycobiome and the nine habitat types were also revealed using Dendrogram analysis, showing that the mycobiome in the samples clustered by habitat type (Figure S1). For example, 24 seawater samples grouped and were isolated from those in the other eight habitats; 8 marine sediment samples clustered together and were also isolated from those in the other samples; and 12 dung samples grouped and were also isolated from those in the other habitats.

**Figure 4:**
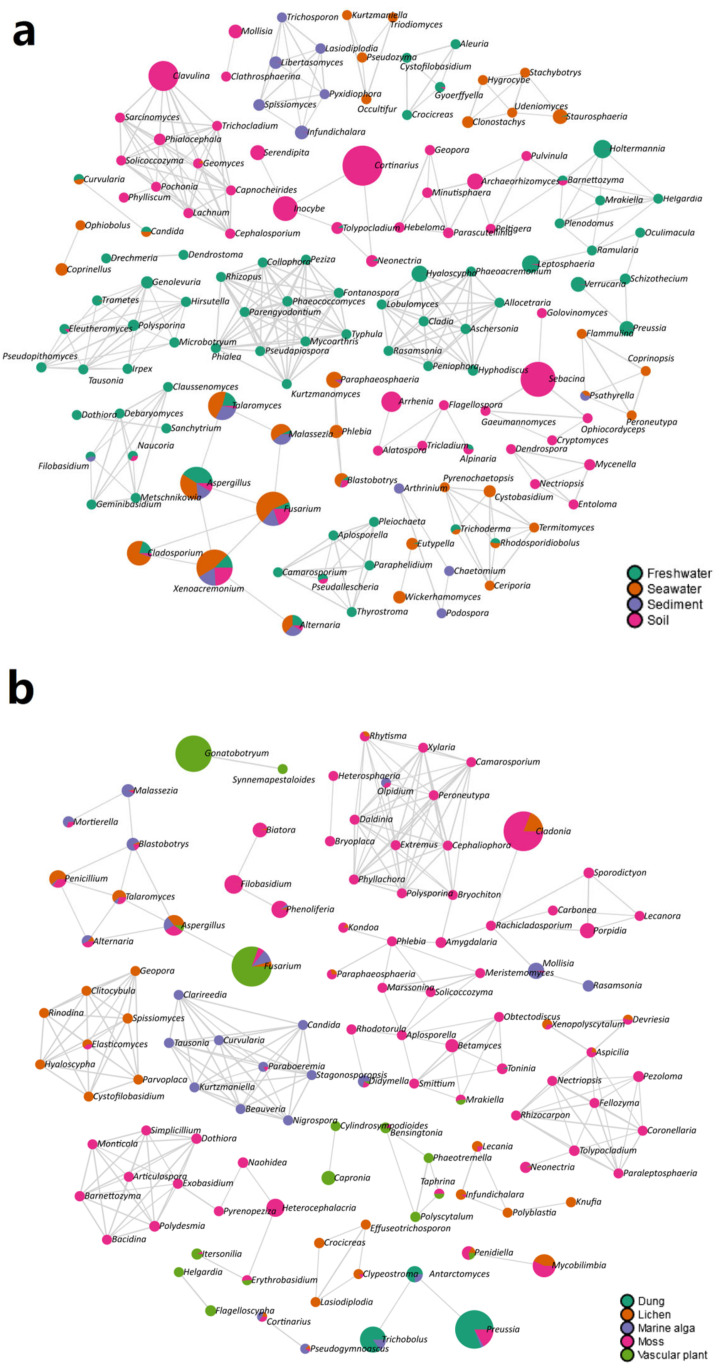
Correlation network analyses showing the correlations of the fungal genera in (**a**) four habitat types (i.e., freshwater, seawater, sediment, and soil) and (**b**) five habitat types (i.e., dung, lichen, marine alga, moss, and vascular plant). Each node represents taxa at the genus level and the edges represent correlations between taxa pairs. The nodes are colored based on habitat types.

The co-occurrence networks demonstrated that there was a strong relationship between the fungal genera and habitat types (Figure 4). For instance, six fungal genera were found in the same module and were present in the sediment, including *Pyxidiophora*, *Trichosporon*, *Libertasomyces*, *Infundichalara*, *Lasiodiplodia*, and *Spissiomyces* (Figure 4a). In addition, nine fungal genera (i.e., *Tausonia*, *Curvularia*, *Clarireedia*, *Stagonosporopsis*, *Beauveria*, *Candida*, *Nigrospora*, *Kurtzmaniella*, and *Paraboeremia*) were in the same module and were harbored in the marine alga (Figure 4b).

**Figure 5:**
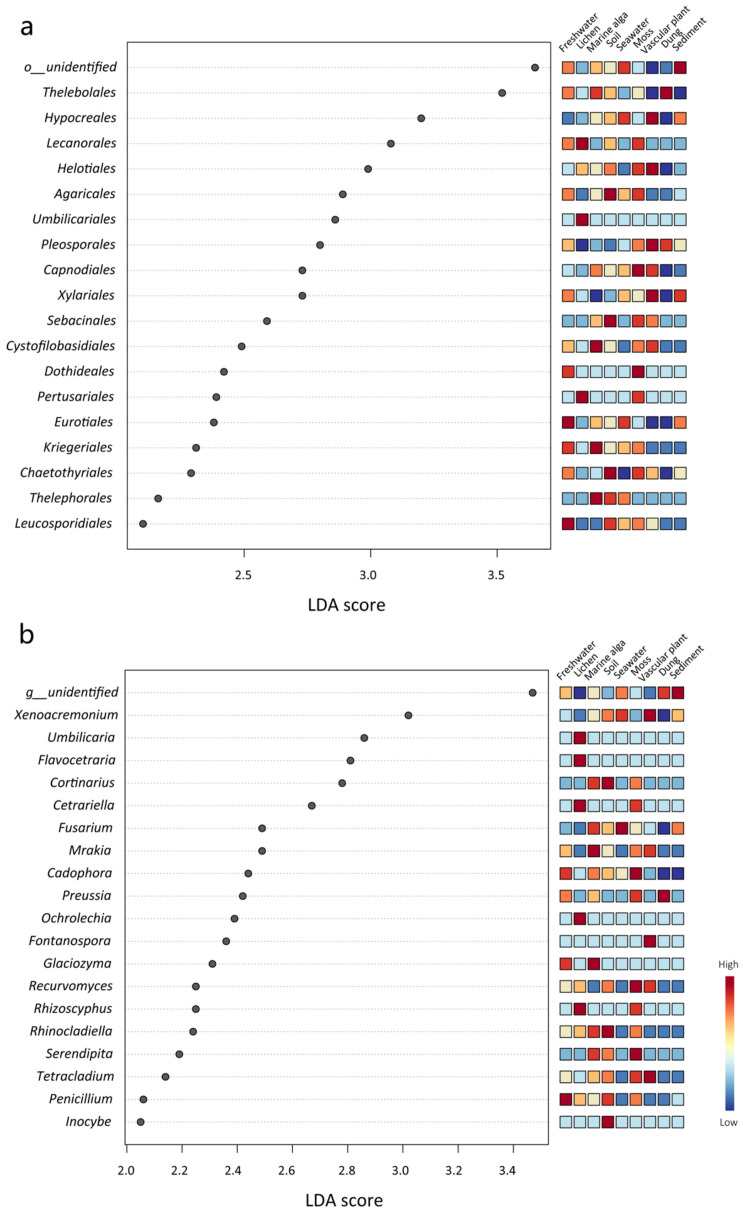
LEfSe analysis showing (**a**) the fungal orders and (**b**) the genera that differed significantly among the nine habitat types. The mini heatmap to the right of the plot indicates whether the taxa are higher (red) or lower (blue) in each group.

The LEfSe analyses showed that some fungal phyla, classes, orders, families, and genera were significantly different among the different habitat types (Figure 5, Figures S2–S4). For example, at the phylum level, *Ascomycota* was significantly abundant in the habitat of vascular plant, *Basidiomycota* was significantly abundant in the soil habitat, and *Chytridiomycota* was significantly abundant in the habitats of marine alga and freshwater (Figure S2). At the order level, *Theleobolales* was significantly abundant in the dung habitat, and *Helotiales* was significantly abundant in the habitat of vascular plant. Moreover, *Agaricales*, *Sebacinales*, and *Chaetothyriales* were significantly abundant in the soil habitat, while *Capnodiales* and *Dothideales* were significantly abundant in the moss habitat (Figure 5a). At the genus level, *Xenoacremonium* and *Fontanospora* were significantly abundant in the habitat of vascular plant; *Mrakia* and *Glaciozyma* were significantly abundant in the habitat of marine alga; *Fusarium* was significantly abundant in the habitat of seawater; *Cortinarius* and *Inocybe* were significantly abundant in the habitat of soil (Figure 5b).

### 3.4. Ecological Role of the Mycobiome in the Nine Habitats

A total of 19 functional guilds and 6 growth forms were discovered in the 114 samples. Table S3 showed the functional assignments of the fungal ASVs identified at the general level. Saprophytic fungi (989 ASVs) dominated the mycobiome in these samples, followed by plant pathogens (185 ASVs), lichenized fungi (116 ASVs), endophytic fungi (45 ASVs), and ectomycorrhizal fungi (41 ASVs).

**Figure 6:**
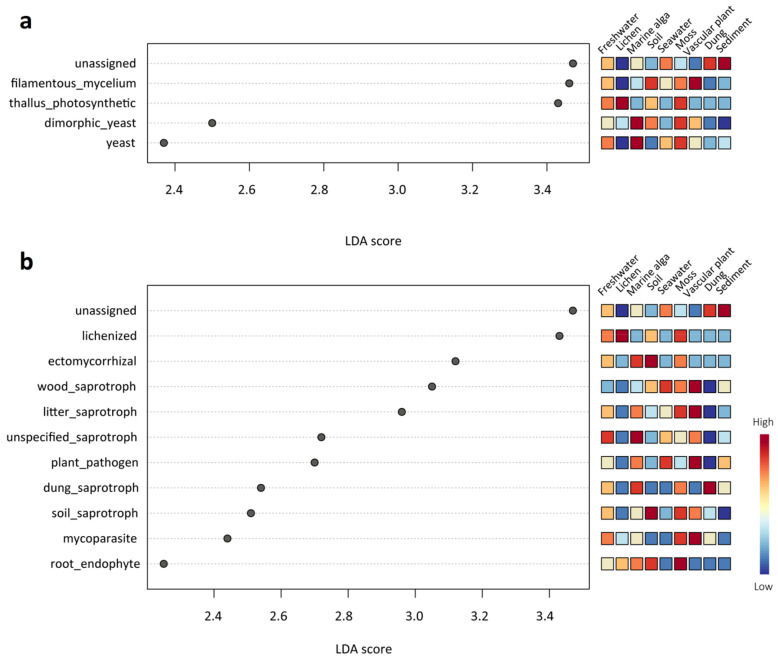
LEfSe analysis showing (**a**) the growth forms and (**b**) the functional guilds that differed significantly among the nine habitat types. The mini heatmap to the right of the plot indicates whether the taxa are higher (red) or lower (blue) in each group.

The results of the ANOSIM tests revealed that the fungal guilds (*R* = 0.50281, *p* < 0.001) and growth forms (*R* = 0.50285, *p* < 0.001) among the nine different habitats differed significantly. Many significant differences in the fungal functional role were also observed among different habitats using LEfSe analysis (Figure 6). In terms of growth forms, dimorphic yeast and yeast were significantly abundant in the marine alga habitat; thallus photosynthetic was significantly abundant in the lichen habitat; and filamentous mycelium was significantly abundant in the vascular plant habitat (Figure 6a). In terms of the ecological guild, lichenized fungi were significantly abundant in the lichen habitat; ectomycorrhizal fungi and soil saprotroph were significantly abundant in the soil habitat; and root endophyte was significantly abundant in the moss habitat (Figure 6b).

The occurrence of 31 fungal species that were considered to be potential human pathogens was determined. The common species included *Alternaria tenuissima* (41 samples in 7 habitats), *Malassezia restricta* (41 samples in 7 habitats), *Aspergillus sydowii* (17 samples in 8 habitats), *Didymella glomerata* (12 samples in 5 habitats), *Filobasidium magnum* (12 samples in 4 habitats), *Cladosporium cladosporioides* (11 samples in 6 habitats), and *Naganishia albida* (10 samples in 3 habitats) (Table S4).

## 4. Discussion

Many studies have indicated that changes in environmental conditions have a significant impact on the fungal communities in the Arctic [4,5,6], and thereby it is urgent for an integrative study to unravel the diversity and functional role of the mycobiome in the Arctic that exists now. Studies on the mycobiome have, to date, largely focused on limited Arctic habitats, and there hasn’t been much research conducted to investigate different habitats at a local scale.

The most prevalent fungal ASVs in the present study were categorized into unidentified phyla, which suggests the dominance of undescribed fungal species. The common phyla identified were *Ascomycota*, *Basidiomycota*, *Chytridiomycota*, *Mortierellomycota*, *Mucoromycota*, and *Rozellomycota*, which were reported in previous 454 pyrosequencing studies [15,17,18,19,22,26]. ASVs belonging to the infrequently reported phyla *Olpidiomycota*, *Monoblepharomycota*, *Zoopagomycota*, *Aphelidiomycota*, and *Kickxellomycota*, were also discovered using Illumina sequencing (Figure 2a). Furthermore, a total of 81 orders, 278 genera, and 261 species were found in this area’s nine habitats. In earlier researches, 28 orders and 95 genera were detectable in Arctic soils using 454 pyrosequencing [15], while 24 orders, 90 genera, and 76 species were detectable in freshwater [26], 28 orders, 51 genera, and 31 species were detectable in vascular plants [22], 40 orders, 78 genera, and 55 species were detectable in lichens [18], and 25 orders, 30 genera, and 30 species were detectable in marine sediments [17]. The results provided a more integrative understanding of the fungal taxonomic diversity in a High Arctic region.

The abundance of some fungal phyla, classes, orders, families, and genera differed significantly among the nine habitats. For example, the order *Agariales* was abundant in the soil habitat, and the order *Helotiales* was abundant in the moss and vascular plant habitats (Figure 2b). The roots of the Arctic vascular plant (*Ericaceae*) host a diverse mycobiome dominated by the order *Helotiales* [45]. In previous 454 pyrosequencing analyses of fungal communities in Arctic mosses, the order *Helotiales* was the dominant component of the communities inhabiting Arctic mosses and vascular plants [19]. In our previous study, a total of 21 macrofungal species belonging to the order *Agaricales* were identified in the Ny-Ålesund Region [46].

In the present study, 19 functional guilds and 6 growth forms were identified, suggesting that the mycobiome in the High Arctic may serve a range of functional roles in various habitats. We found saprophytic fungi (989 ASVs) dominated the fungal community in this High Arctic region. Saprotrophic fungi increase the availability of soil nutrients by actively decomposing organic matter through the secretion of a range of hydrolases, such as proteinases, cellulases, and laccases [47]. Interestingly, many fungal functional groups (i.e., guilds and growth forms) were also significantly different among the nine habitats (Figure 6), which was inconsistent with the results of the taxonomic groups (i.e., order and genera) (Figure 5). For example, ectomycorrhizal fungi (order *Agaricales*, genera *Cortinarius* and *Inocybe*) were more abundant in the soil habitat, lichenized fungi (order *Lecanorales, Umbiliariales*, and *Pertusariales*, genera *Flavocetraria*, *Cetrariella, Umbilicaria*, and *Ochrolechia*) were more abundant in the lichen habitat, root endophytes (genera *Serendipita*) were more abundant in the moss habitat, and yeasts (order *Cystofilobasidiales*, genera *Glaciozyma* and *Mrakia*) were more abundant in the marine alga habitat. Ectomycorrhizal fungi, which provide plants with mineral nutrients and water from the soil, are necessary for Arctic vascular plants to survive (e.g., *Betula*, *Dryas*, and *Salix*) [48]. The root endophytic fungus *Serendipita* belongs to the order *Sebacinales* and can alter the root architecture and improve phosphorus (P) and zinc (Zn) uptake [49]. Therefore, habitat filtering may affect the colonization of fungi, with some fungal species being chosen based on their functional characteristics [13].

The habitat specificity of the mycobiome at the local scale may be attributed to variations in environmental factors (such as the physicochemical proprieties, nutritional levels, antagonistic factors, etc.). There are many unmeasured environmental factors in the nine habitats of the Ny-Ålesund Region, which may affect the diversity and composition of the mycobiome in this region. In Western Greenland (Low Arctic), varied vegetation types and environmental conditions, such as pH and water content, were shown to be associated with changes in the fungal community composition and functional role [28].

The composition of the mycobiome may be affected by climate change directly or indirectly [4]. As a result of climate warming, the melting of permafrost can alter aquatic fungal diversity and fungal community composition through interactions with carbon released from sediments [25]. Some fungi can be utilized as bioindicators of changes taking place in the Arctic environment, such as the genus *Cladosporium* [50]. In addition, permafrost may be a reservoir of potential fungal pathogens [51], which may be released as a result of climate warming. Previous research has shown the presence of potential fungal pathogens in Arctic environments, such as snow, ice, meltwater, and air. These pathogens include *Aureobasidium melanogenum*, *Naganishia albida*, and *Rhodotorula mucilaginosa*, which can grow at 37 °C, exhibit β-hemolytic activity, and are resistant to azoles and echinocandins [31]. Potential human fungal pathogens (31 species) were discovered in the present study, according to the Atlas of Clinical Fungi [44]. Among the potential fungal pathogens (31 species) observed in the present study, seven fungal species are listed as Biosafety Level 2 (BSL-2), including *Aspergillus fumigatus*, *Fusarium solani*, *Malassezia globosa*, *Rhizopus arrhizus*, *Sarocladium kiliense*, *Trichosporon asahii*, and *Wickerhamomyces anomalus*. To determine the fungal virulence detected in the Arctic habitats, which is essential for the pathogenicity of fungi, additional analyses of fungal isolates are required in further studies.

## 5. Conclusions

This integrative study reveals the diversity and distribution of the mycobiome in the nine distinct habitats and highlights their functional role in the High Arctic region. We also found that, while the mycobiome in distinct habitats was related locally through dispersal, habitat specialization rather than overlap drove the mycobiome distribution in the High Arctic region. This suggests that habitat filtering is a significant determinant of fungal assemblages in this Arctic region. Our data may lay a foundation for speculation on the changes in the mycobiome in different habitats due to climate warming in the High Arctic regions. 

## Figures and Tables

**Table 1:** ANOSIM results for the pairwise comparisons of the similarity among the nine habitats.

Habitat Type	Soil	Freshwater	Seawater	Lichen	Sediment	Dung	Marine alga	Moss	Vascular Plant
Soil	-	0.67664 ***	0.84645 ***	0.48733 ***	0.97115 ***	0.97516 ***	0.47797 **	0.37873 ***	0.58004 ***
Freshwater	0.67664 ***	-	0.9078 ***	0.77907 ***	0.97385 ***	1 ***	0.76711 **	0.65965 ***	0.94234 ***
Seawater	0.84645 ***	0.9078 ***	-	0.84193 ***	0.90845 ***	1 ***	0.94132 ***	0.92049 ***	0.99311 ***
Lichen	0.48733 ***	0.77907 ***	0.84193 ***	-	0.82897 ***	0.40703 ***	0.43981 **	0.50916 ***	0.48796 ***
Sediment	0.97115 ***	0.97385 ***	0.90845 ***	0.82897 ***	-	1 ***	0.94868 ***	0.96906 ***	1 ***
Dung	0.97516 ***	1 ***	1 ***	0.40703 ***	1 ***	-	0.90614 **	0.95968 ***	0.92961 ***
Marine alga	0.47797 **	0.76711 **	0.94132 ***	0.43981 **	0.94868 ***	0.90614 **	-	0.61455 ***	0.42719 **
Moss	0.37873 ***	0.65965 ***	0.92049 ***	0.50916 ***	0.96906 ***	0.95968 ***	0.61455 ***	-	0.60594 ***
Vascular plant	0.58004 ***	0.94234 ***	0.99311 ***	0.48796 ***	1 ***	0.92961 ***	0.42719 **	0.60594 ***	-

** 0.001 < *p* ≤ 0.01, *** *p* ≤ 0.001.

## Data Availability

All the raw sequence files are available in the National Center for Biotechnology Information under the BioProject ID PRJNA746944.

## Supplementary Materials

The following are available online at https://www.mdpi.com/article/10.3390/jof9040437/s1. Figure S1: Dendrogram showing fungal communities in the 114 samples collected from the nine habitats; Figure S2: LEfSe analysis showing the fungal phyla that are significantly different among the nine habitats of the Ny-Ålesund Region; Figure S3: LEfSe analysis showing the fungal classes that are significantly different among the nine habitats of the Ny-Ålesund Region; Figure S4: LEfSe analysis showing the fungal families that are significantly different among the nine habitats of the Ny-Ålesund Region; Table S1: Information on the 114 samples collected from the Ny-Ålesund Region; Table S2: Information on the 11,850 fungal ASVs in the 114 samples collected from the Ny-Ålesund Region; Table S3: Information on the 10,419 fungal ASVs in the 114 samples collected from the Ny-Ålesund Region; Table S4: An overview of potentially pathogenic fungi found in the nine habitats from the Ny-Ålesund Region.
