# Supplementary material for: An Integrative Study of Mycobiome in Different Habitats from a High Arctic Region: Diversity, Distribution, and Functional Role
Source: J Fungi (Basel). 2023 Apr 3;9(4):437. doi: 10.3390/jof9040437 (PMC10144742; doi:10.3390/jof9040437)
Supplement: Supplementary file 1 [file jof-09-00437-s001.zip › Figs. S1 to S4.pdf]

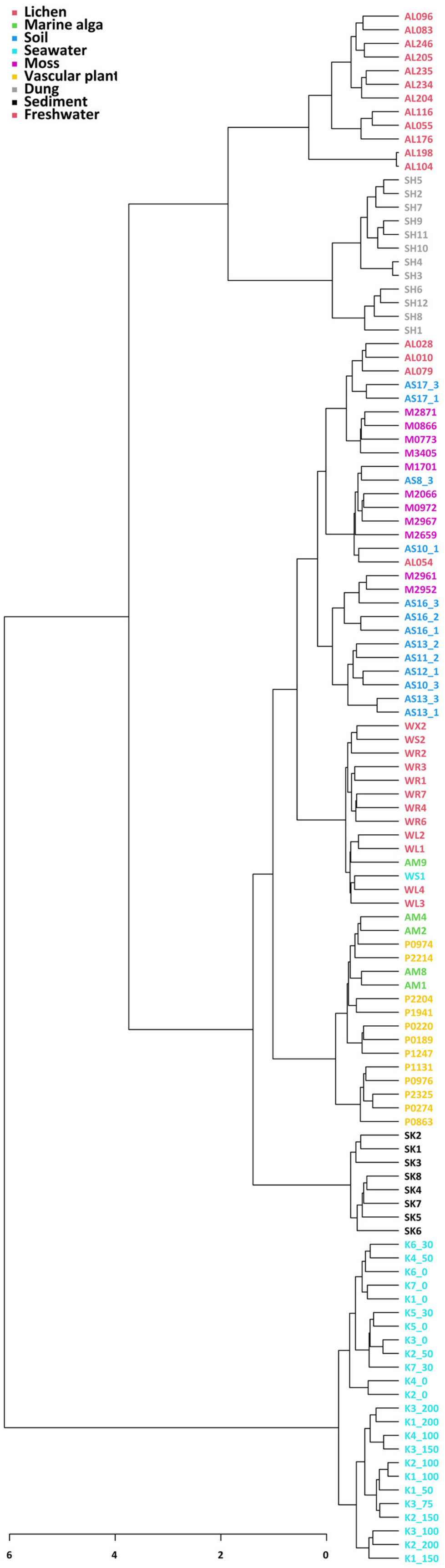

Figure S1. Dendrogram showing fungal communities in the 114 samples collected from the nine habitats.

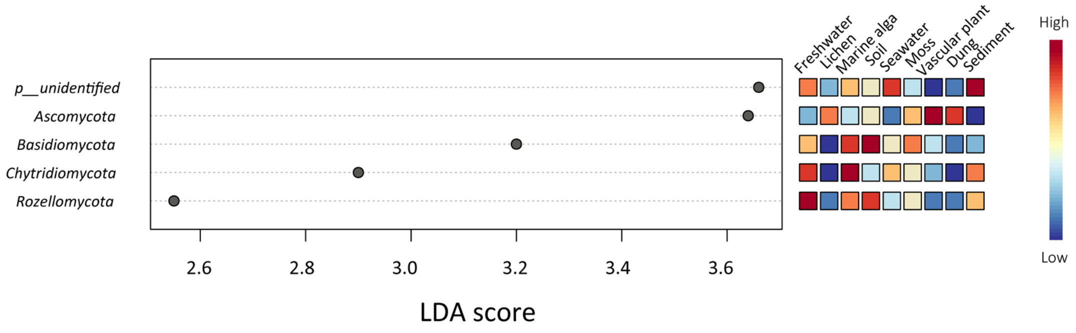

Figure S2. LEfSe analysis showing the fungal phyla that are significantly different among the nine habitats of the Ny-Ålesund Region. Significant phyla are ranked by their LDA scores (x axis). The mini heatmap to the right of the plot indicates whether the phyla are higher (red) or lower (blue) in each habitat.

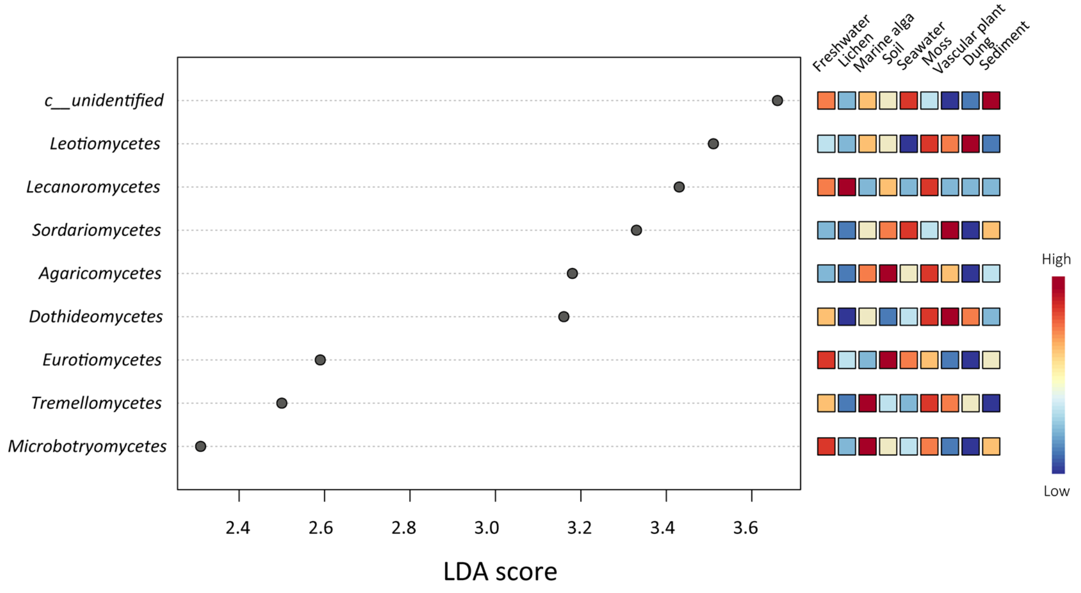

Figure S3. LEfSe analysis showing the fungal classes that are significantly different among the nine habitats of the Ny-Ålesund Region. Significant classes are ranked by their LDA scores (x axis). The mini heatmap to the right of the plot indicates whether the classes are higher (red) or lower (blue) in each habitat.

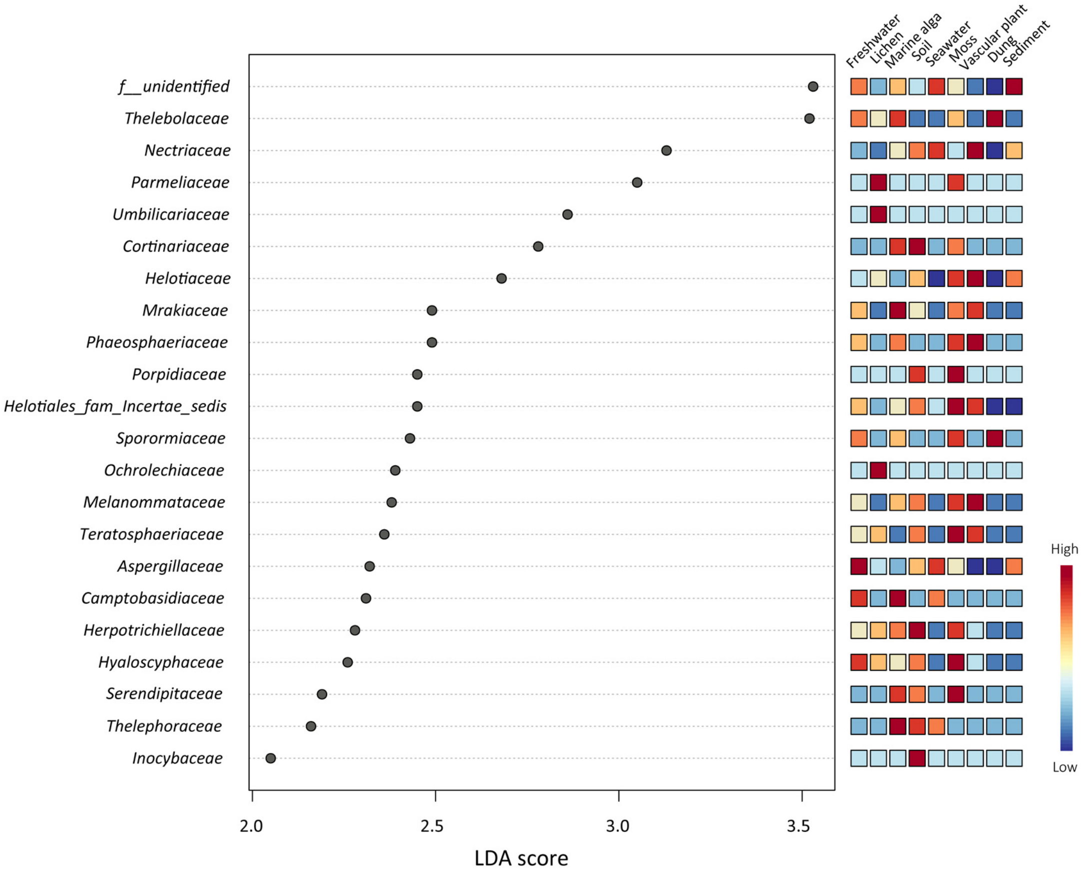

Figure S4. LEfSe analysis showing the fungal families that are significantly different among the nine habitats of the Ny-Ålesund Region. Significant families are ranked by their LDA scores (x axis). The mini heatmap to the right of the plot indicates whether the families are higher (red) or lower (blue) in each habitat.
