# Supplementary material for: An Integrative Study of Mycobiome in Different Habitats from a High Arctic Region: Diversity, Distribution, and Functional Role
Source: J Fungi (Basel). 2023 Apr 3;9(4):437. doi: 10.3390/jof9040437 (PMC10144742; doi:10.3390/jof9040437)
Supplement: Supplementary file 1 [file jof-09-00437-s001.zip › Table S1.pdf]

Table S1. Information on the 114 samples collected from the Ny-Ålesund Region.

no.	Sampling code	Habitat type	Raw reads	Trimmed reads	Subsampled reads	Number of fungal ASVs	NCBI SRA Number
1	AL010	Lichen (*Stereocaulon alpinum*)	31378	26452	14866	164	In this study
2	AL028	Lichen (*Stereocaulon alpinum*)	36560	31053	14866	124	In this study
3	AL054	Lichen (*Peltigera canina*)	32898	23852	14866	95	In this study
4	AL055	Lichen (*Umbilicaria arctica*)	39026	30328	14866	8	In this study
5	AL079	Lichen (*Ochrolechia frigida*)	38040	31923	14866	149	In this study
6	AL083	Lichen (*Ochrolechia frigida*)	33446	29367	14866	22	In this study
7	AL096	Lichen (*Cetrariella delisei*)	44444	36697	14866	24	In this study
8	AL104	Lichen (*Peltigera canina*)	34385	32368	14866	2	In this study
9	AL116	Lichen (*Umbilicaria arctica*)	42830	32923	14866	2	In this study
10	AL176	Lichen (*Umbilicaria arctica*)	43375	33111	14866	10	In this study
11	AL198	Lichen (*Peltigera canina*)	35644	30596	14866	2	In this study
12	AL204	Lichen (*Flavocetraria nivalis*)	37647	32422	14866	14	In this study
13	AL205	Lichen (*Ochrolechia frigida*)	40234	29452	14866	17	In this study
14	AL234	Lichen (*Cetrariella delisei*)	41094	33805	14866	2	In this study
15	AL235	Lichen (*Flavocetraria nivalis*)	37295	32290	14866	8	In this study
16	AL246	Lichen (*Flavocetraria nivalis*)	44155	35156	14866	19	In this study
17	AM1	Marine alga	38502	35201	14866	44	In this study
18	AM2	Marine alga	33101	30060	14866	56	In this study
19	AM4	Marine alga	43292	35081	14866	45	In this study
20	AM8	Marine alga	31214	27537	14866	29	In this study
21	AM9	Marine alga	41306	29396	14866	298	In this study
22	AS10_1	Soil	35633	28119	14866	108	PRJNA448984
23	AS10_3	Soil	34170	31171	14866	83	PRJNA448984
24	AS11_2	Soil	33348	26533	14866	25	PRJNA448984
25	AS12_1	Soil	32210	28620	14866	99	PRJNA448984
26	AS13_1	Soil	39695	34123	14866	130	PRJNA448984
27	AS13_2	Soil	33299	27974	14866	39	PRJNA448984
28	AS13_3	Soil	34458	30746	14866	139	PRJNA448984
29	AS16_1	Soil	32937	25566	14866	62	PRJNA448984
30	AS16_2	Soil	41723	38026	14866	87	PRJNA448984
31	AS16_3	Soil	32930	24453	14866	82	PRJNA448984
32	AS17_1	Soil	33729	28177	14866	301	PRJNA448984
33	AS17_3	Soil	42012	33805	14866	203	PRJNA448984
34	AS08_3	Soil	33116	27093	14866	50	PRJNA448984
35	K1_0	Seawater (0 m in Kongsfjorden)	36765	29002	14866	167	In this study
36	K1_100	Seawater (-100 m in Kongsfjorden)	20039	17636	14866	138	In this study
37	K1_150	Seawater (-100 m in Kongsfjorden)	26230	22515	14866	203	In this study
38	K1_200	Seawater (-100 m in Kongsfjorden)	32318	27110	14866	191	In this study
39	K1_50	Seawater (0 m in Kongsfjorden)	25124	19223	14866	138	In this study
40	K2_0	Seawater (0 m in Kongsfjorden)	19836	16701	14866	54	In this study
41	K2_100	Seawater (-100 m in Kongsfjorden)	40578	30973	14866	168	In this study

42	K2_150	Seawater (-150 m in Kongsfjorden)	31716	23645	14866	208	In this study
43	K2_200	Seawater (-200 m in Kongsfjorden)	20254	14896	14866	171	In this study
44	K2_50	Seawater (-50 m in Kongsfjorden)	28531	15090	14866	112	In this study
45	K3_0	Seawater (0 m in Kongsfjorden)	31892	27075	14866	98	In this study
46	K3_100	Seawater (-100 m in Kongsfjorden)	20503	15660	14866	142	In this study
47	K3_150	Seawater (-150 m in Kongsfjorden)	21177	16945	14866	126	In this study
48	K3_200	Seawater (-200 m in Kongsfjorden)	31448	22851	14866	198	In this study
49	K3_75	Seawater (-75 m in Kongsfjorden)	37680	29683	14866	182	In this study
50	K4_0	Seawater (0 m in Kongsfjorden)	47145	39504	14866	97	In this study
51	K4_100	Seawater (-100 m in Kongsfjorden)	22070	18009	14866	143	In this study
52	K4_50	Seawater (-50 m in Kongsfjorden)	25372	20558	14866	207	In this study
53	K5_0	Seawater (0 m in Kongsfjorden)	35640	28872	14866	155	In this study
54	K5_30	Seawater (-30 m in Kongsfjorden)	29135	21058	14866	124	In this study
55	K6_0	Seawater (0 m in Kongsfjorden)	37455	30021	14866	176	In this study
56	K6_30	Seawater (-30 m in Kongsfjorden)	28245	23975	14866	175	In this study
57	K7_0	Seawater (0 m in Kongsfjorden)	40346	28706	14866	170	In this study
58	K7_30	Seawater (-30 m in Kongsfjorden)	28187	24345	14866	128	In this study
59	WS1	Seawater (0 m near shore)	31052	21863	14866	210	In this study
60	M0773	Moss (*Andreaea rupestris*)	33629	29888	14866	320	PRJNA448984
61	M0866	Moss (*Cynodontium* sp.)	35349	27909	14866	186	PRJNA448984
62	M0972	Moss (*Sanionia uncinata*)	36821	18224	14866	187	PRJNA448984
63	M1701	Moss (*Polytrichastrum alpinum*)	41235	37820	14866	186	PRJNA448984
64	M2066	Moss (*Sanionia uncinata*)	33053	21647	14866	109	PRJNA448984
65	M2659	Moss (*Sarmentypnum* sp.)	39285	29108	14866	198	In this study
66	M2871	Moss (*Andreaea rupestris*)	36469	22346	14866	275	PRJNA448984
67	M2952	Moss (*Bryum purpurascens*)	41500	38877	14866	82	PRJNA448984
68	M2961	Moss (*Bryum purpurascens*)	35261	33473	14866	43	PRJNA448984
69	M2967	Moss (*Polytrichastrum alpinum*)	36176	33535	14866	132	PRJNA448984
70	M3405	Moss (*Cynodontium* sp.)	44121	35080	14866	242	PRJNA448984
71	P0189	Vascular plant (*Silene acaulis*)	31087	28498	14866	26	PRJNA448984
72	P0220	Vascular plant (*Carex misandra*)	32440	27713	14866	22	PRJNA448984
73	P0214	Vascular plant (*Saxifraga oppositifolia*)	31678	27960	14866	73	PRJNA448984
74	P0863	Vascular plant (*Silene acaulis*)	41466	21086	14866	83	PRJNA448984
75	P0974	Vascular plant (*Silene acaulis*)	37773	31991	14866	18	PRJNA448984
76	P0976	Vascular plant (*Saxifraga cespitosa*)	35335	31511	14866	61	PRJNA448984
77	P1131	Vascular plant (*Saxifraga cespitosa*)	35047	28745	14866	74	PRJNA448984
78	P1247	Vascular plant (*Saxifraga cespitosa*)	40006	35876	14866	14	PRJNA448984
79	P1941	Vascular plant (*Cassiopetetragona*)	31426	26283	14866	26	PRJNA448984
80	P2204	Vascular plant (*Cassiopetetragona*)	38160	33670	14866	61	PRJNA448984
81	P2214	Vascular plant (*Saxifraga oppositifolia*)	30698	27540	14866	10	PRJNA448984
82	P2325	Vascular plant (*Saxifraga oppositifolia*)	33908	30221	14866	56	PRJNA448984
83	SH1	Dung of reindeer	33748	31502	14866	10	In this study
84	SH2	Dung of reindeer	37121	23499	14866	27	In this study
85	SH3	Dung of reindeer	35637	31843	14866	15	In this study

86	SH4	Dung of reindeer	33625	31337	14866	17	In this study
87	SH5	Dung of reindeer	40706	38059	14866	22	In this study
88	SH6	Dung of reindeer	30281	19204	14866	25	In this study
89	SH7	Dung of reindeer	41314	38510	14866	33	In this study
90	SH8	Dung of reindeer	41511	38724	14866	19	In this study
91	SH9	Dung of reindeer	33622	30743	14866	27	In this study
92	SH10	Dung of reindeer	44661	41624	14866	27	In this study
93	SH11	Dung of reindeer	33266	29604	14866	17	In this study
94	SH12	Dung of reindeer	41675	38396	14866	28	In this study
95	SK1	Sediment in Kongsfjorden	20244	15370	14866	95	In this study
96	SK2	Sediment in Kongsfjorden	39990	33694	14866	229	In this study
97	SK3	Sediment in Kongsfjorden	33109	22756	14866	104	In this study
98	SK4	Sediment in Kongsfjorden	43377	27623	14866	221	In this study
99	SK5	Sediment in Kongsfjorden	53232	45229	14866	273	In this study
100	SK6	Sediment in Kongsfjorden	35628	31335	14866	217	In this study
101	SK7	Sediment in Kongsfjorden	53230	22083	14866	191	In this study
102	SK8	Sediment in Kongsfjorden	55344	45495	14866	250	In this study
103	WL1	Freshwater (pond)	82706	71718	14866	209	PRJNA448984
104	WL2	Freshwater (pond)	35062	30403	14866	186	PRJNA448984
105	WL3	Freshwater (pond)	53644	44803	14866	251	In this study
106	WL4	Freshwater (pond)	33155	28852	14866	191	In this study
107	WR1	Freshwater (stream)	77322	64461	14866	681	PRJNA448984
108	WR2	Freshwater (stream)	105159	97477	14866	441	PRJNA448984
109	WR3	Freshwater (stream)	60143	53249	14866	537	PRJNA448984
110	WR4	Freshwater (stream)	30226	19784	14866	349	PRJNA448984
111	WR6	Freshwater (stream)	41037	31761	14866	302	PRJNA448984
112	WR7	Freshwater (stream)	39527	34962	14866	284	PRJNA448984
113	WS2	Freshwater (estuary)	43758	37887	14866	450	PRJNA448984
114	WX2	Freshwater (ice)	42491	35226	14866	225	PRJNA448984
