# Supplementary material for: An Integrative Study of Mycobiome in Different Habitats from a High Arctic Region: Diversity, Distribution, and Functional Role
Source: J Fungi (Basel). 2023 Apr 3;9(4):437. doi: 10.3390/jof9040437 (PMC10144742; doi:10.3390/jof9040437)
Supplement: Supplementary file 1 [file jof-09-00437-s001.zip › Table S4.pdf]

Table S4. An overview of potentially pathogenic fungi found in the nine habitats from the Ny-Ålesund Region.

Fungal species	Soil	Seawater	Freshwater	Marine alga	Sediment	Vascular plant	Lichen	Moss	Dung	Total
Alternaria tenuissima	3	20	7	2	3	-	3	3	-	41
Aspergillus caesiellus	-	-	2	-	1	-	-	-	-	3
Aspergillus fumigatus	-	-	1	-	-	-	-	-	-	1
Aspergillus penicillioides	1	2	1	1	-	-	1	1	-	7
Aspergillus restrictus	-	-	-	1	-	-	-	-	-	1
Aspergillus sydowii	1	3	4	1	2	1	1	4	-	17
Candida parapsilosis	-	-	1	1	-	-	-	-	-	2
Ceriporia lacerata	-	1	-	-	-	-	-	-	-	1
Cladosporium cladosporioides	1	6	1	1	-	-	1	1	-	11
Cladosporium sphaerospermum	-	1	-	-	-	-	-	-	-	1
Coprinellus radians	-	1	-	-	-	-	-	-	-	1
Curvularia lunata	-	-	1	1	-	-	-	-	-	2
Cystofilobasidium macerans	-	-	-	-	-	-	1	-	-	1
Didymella glomerata	1	-	4	1	-	1	-	5	-	12
Filobasidium magnum	-	-	1	-	1	-	1	9	-	12
Fusarium solani	1	-	1	1	-	1	1	-	-	5
Malassezia globosa	-	2	1	-	-	-	-	-	-	3
Malassezia restricta	1	24	6	2	3	-	1	4	-	41
Meyerozyma guilliermondii	-	1	-	-	-	-	-	-	-	1
Naganishia albida	-	-	-	1	-	-	-	7	2	10
Papiliotrema flavescens	1	-	-	-	-	-	-	-	-	1
Parengyodontium album	-	-	1	-	-	-	-	-	-	1
Penicillium aurantiogriseum	1	-	-	-	-	-	-	-	-	1
Penicillium citrinum	-	-	-	-	-	-	-	1	-	1
Penicillium oxalicum	-	-	3	-	-	-	-	-	-	3
Pseudogymnoascus destructans	4	-	4	-	-	-	-	-	-	8
Rhizopus arrhizus	-	-	1	-	-	-	-	-	-	1
Rhodotorula mucilaginosa	-	1	1	-	1	-	-	-	-	3
Sarocladium kiliense	-	-	2	2	1	-	-	-	-	5
Trichosporon asahii	-	-	-	-	1	-	-	-	-	1
Wickerhamomyces anomalus	-	1	-	-	-	-	-	-	-	1

The number in columns indicate the number of samples in which fungal species was detected.
